# Understanding Loneliness Through Analysis of Twitter and Reddit Data: Comparative Study

**DOI:** 10.2196/49464

**Published:** 2025-03-14

**Authors:** Hurmat Ali Shah, Mowafa Househ

**Affiliations:** 1 Hamad Bin Khalifa University Doha Qatar

**Keywords:** health informatics, loneliness informatics, loneliness theory, health effects, loneliness interventions, social media, lonely, loneliness, isolation, mental health, natural language processing, tweet, tweets, comparative analysis

## Abstract

**Background:**

Loneliness is a global public health issue contributing to a variety of mental and physical health issues. It increases the risk of life-threatening conditions and contributes to the burden on the economy in terms of the number of productive days lost. Loneliness is a highly varied concept, which is associated with multiple factors.

**Objective:**

This study aimed to understand loneliness through a comparative analysis of loneliness data on Twitter and Reddit, which are popular social media platforms. These platforms differ in terms of their use, as Twitter allows only short posts, while Reddit allows long posts in a forum setting.

**Methods:**

We collected global data on loneliness in October 2022. Twitter posts containing the words “lonely,” “loneliness,” “alone,” “solitude,” and “isolation” were collected. Reddit posts were extracted in March 2023. Using natural language processing techniques (valence aware dictionary for sentiment reasoning [VADER] tool from the natural language toolkit [NLTK]), the study identified and extracted relevant keywords and phrases related to loneliness from user-generated content on both platforms. The study used both sentiment analysis and the number of occurrences of a topic. Quantitative analysis was performed to determine the number of occurrences of a topic in tweets and posts, and overall meaningful topics were reported under a category.

**Results:**

The extracted data were subjected to comparative analysis to identify common themes and trends related to loneliness across Twitter and Reddit. A total of 100,000 collected tweets and 10,000 unique Reddit posts, including comments, were analyzed. The results of the study revealed the relationships of various social, political, and personal-emotional themes with the expression of loneliness on social media. Both platforms showed similar patterns in terms of themes and categories of discussion in conjunction with loneliness-related content. Both Reddit and Twitter addressed loneliness, but they differed in terms of focus. Reddit discussions were predominantly centered on personal-emotional themes, with a higher occurrence of these topics. Twitter, while still emphasizing personal-emotional themes, included a broader range of categories. Both platforms aligned with psychological linguistic features related to the self-expression of mental health issues. The key difference was in the range of topics, with Twitter having a wider variety of topics and Reddit having more focus on personal-emotional aspects.

**Conclusions:**

Reddit posts provide detailed insights into data about the expression of loneliness, although at the cost of the diversity of themes and categories, which can be inferred from the data. These insights can guide future research using social media data to understand loneliness. The findings provide the basis for further comparative investigation of the expression of loneliness on different social media platforms and online platforms.

## Introduction

Loneliness not only affects quality of life but also leads to other mental health issues, thus burdening the public health service system. The monetary loss as a result of loneliness is estimated to be between US $8074.80 and US $12,077.70 per person per year in the United Kingdom [1]. The monetary cost of lost days and loss in productivity is estimated to be US $3.14 billion per year for employees in the United Kingdom. Loneliness is shown to be associated with a high risk for multiple health conditions such as physical and mental health issues, dementia, and early mortality [2].

Loneliness is formally defined as, “the unpleasant experience that occurs when a person’s network of social relationships is deficient in some important way, quantitatively or qualitatively” [3]. Loneliness, thus, is the perceived and subjective dissonance between one’s desired and actual social contacts and relationships. It is difficult to know whether a person is lonely when there is no direct reporting by the person, as loneliness is a very subjective phenomenon. As opposed to social isolation, which is objective and points to a lack of any social connection, loneliness is hard to identify. Loneliness also follows a U-shaped curve demographically, that is, young and old people are the loneliest [4]. Because social media and technology are more frequently used by the younger generation, they are helpful for analyzing loneliness. A previous report provided a theoretical explication of loneliness, loneliness informatics, and the gaps in research on the topic of loneliness informatics, that is, the application of informatics tools to study loneliness [5].

The causes of mental health issues can vary from genetic factors to social and economic factors, and might involve immediate family or finding meaning in life. The result can be withdrawal from human bonds and touch. Loneliness can be addressed through different interventions. However, there is a need to understand the prevalence of loneliness to devise technology-based and community-oriented strategies. Technology may have resulted in fragmented and individualized existence, but it can also be a great healer. The rise of social media has transformed the way we interact with others, offering new opportunities for social connection and communication. Loneliness is a common experience that can have negative effects on mental and physical health, and social media use has been implicated as a potential contributor to loneliness.

To better understand loneliness, this study performed a comparative analysis of Twitter and Reddit data. The aim of this study was to analyze data from these 2 different social media platforms to identify the topics and themes highly associated with the mention of loneliness. We aimed to identify the associations of socioeconomic, political, and personal-psychological factors with the feeling and expression of loneliness. We were not interested in finding out how many people are lonely. We wanted to assess what kind of expression is associated with loneliness. Both platforms are popular social media sites that allow users to post and interact with others, but they differ in terms of their user base and content focus. Twitter is a micro-blogging site with a broad user base that covers a wide range of topics, while Reddit is a forum-based site with niche communities focused on specific topics.

Loneliness is a subjective experience. Social media has become one of the best sources to study loneliness because loneliness must be studied by self-reporting of this subjective feeling. Understanding the nature of social media platforms viz-a-viz the kind of expression about loneliness is important. This study is a step toward understanding the difference in how loneliness is expressed via different social media platforms. The aim is not to conceptualize loneliness per se as this is beyond the scope of this study, but our analysis will provide insights into what terms, topics, and categories occur more frequently in the mention of loneliness.

The aim of the study is not to discuss the qualitative difference between Reddit and Twitter. The difference between both platforms will stand for all topics of analysis. We do not claim to present the differences between the 2 platforms, which is beyond the scope of this paper. Moreover, discussing the differences between different social media platforms may not be of research interest. The research interest is to assess whether the expression of loneliness varies on different social media platforms. We found that there was some variance. The actual reasons may not be known as we are not aware of any research that mentions why people prefer one social media platform over another. Using natural language processing techniques, we analyzed user-generated content on both platforms to identify and extract keywords and phrases related to loneliness. We then compared the prevalence and nature of loneliness-related content on Twitter and Reddit to identify common themes and trends related to loneliness across the 2 platforms. We wanted to understand the topics mostly associated with loneliness and their frequency of occurrence with the mention of loneliness. Before carrying out this analysis, sentiment analysis of tweets was performed through the valence aware dictionary for sentiment reasoning (VADER) tool from the natural language toolkit (NLTK) [6]. The research questions were as follows:

1. What topics and themes can be identified from the data collected on the expression of loneliness from social media? It is important to understand what the data covers and then to carry out an objective comparison.
2. Are there differences in the expression of loneliness on different social media platforms?
3. How the topics and themes associated with loneliness across social media platforms can be divided into various categories?

Social media data with public access are available for Twitter and Reddit. Facebook, a popular social media platform, does not provide access to information based on key terms. As of October 2023, Facebook provides access to an Ad Targeting dataset. Quora, which is a blog-based social media platform, does not have any public application programming interface (API) to provide access to its information. Reddit and Twitter were selected as their data were publicly available when we started collecting data.

Reddit and Twitter as well as other social media platforms can have their own audience, and it cannot be said with any certainty why people use or prefer one platform over the other. This paper does not provide an answer to this question. The characteristics of each social media platform are broadly true for any kind of data extracted from them. In this study, we wanted to investigate whether the expression of loneliness varies on different social media platforms. The objective reasons or causal reasons for the differences are beyond the scope of this study.

Several systematic and scoping reviews have been performed to better understand loneliness. Understanding loneliness theoretically and evaluating its relation to mental health have been the topics of several studies [7]. Theoretical studies on loneliness have established the negative effects of loneliness. Similarly, from the health informatics perspective, there have been multiple systematic reviews of the connection between loneliness and mental health [8]. Moreover, there have been studies dealing with the application of technology-based interventions to cope with loneliness [5].

A comparative methodology has been used to compare countries in terms of the parameters of public health [9,10]. Although this study does not involve a country-wise comparative analysis, it is pertinent to note that a comparative analysis of different topics is an established practice. Foufi et al [11] used Reddit as a source to mine textual information about chronic diseases, the entities for the expression of diseases and conditions, and their relations. Similarly, Reddit was used by Schrading et al [12] for an analysis of domestic abuse. They developed a classifier based on the data available on Reddit to classify whether a post is about domestic abuse. Twitter data were analyzed by Tsai and Wang [13] to evaluate people’s attitudes toward public health policies. The use of Twitter to analyze mental health or other public health conditions during COVID-19 is a prevalent approach, with studies focusing on a range of issues from insights into the pandemic [14] to analyzing social network data [15] and discerning emotions about COVID-19 [16].

Comparison of social media data for different purposes has been carried out in the literature because each social media platform is associated with a particular kind of user and a particular mode of expression. Cinelli et al [17] used Gab, Facebook, Reddit, and Twitter to assess the difference of opinion across different platforms. Curiskis et al [18] focused specifically on Twitter and Reddit for document clustering and topic modeling, while Gozzi et al [19] performed a comparative analysis of media coverage on Reddit and Wikipedia. Similarly, both Twitter and Reddit were used to monitor the spread of hateful content in the wake of COVID-19 [20].

This study builds on literature reviews involving a comparative analysis of social media and other such comparative techniques to better understand loneliness. This study attempts to assess how loneliness is expressed on Reddit and Twitter. The reason for this analysis is to clarify the roles of these platforms in research on loneliness. As social media can provide an intimate perspective on the experience and expression of the feeling of loneliness, understanding how different social media can contribute to research on loneliness is important. Loneliness is a subjective experience, and analysis of social media data can play a fundamental role in informing strategies to counter loneliness and understand its dynamics. This study attempts to clarify how different social media platforms can be used to understand loneliness. The findings of this study will be helpful in devising a large framework using social media for understanding loneliness.

## Methods

### Ethical Considerations

This study was exempt from seeking ethical approval as it analyzed publicly available online content. The study did not use the identity of the people from the data but provided an overall picture based on opinions expressed publicly. Data were collected from the public domain, and the data analysis involved aggregate terms, which were not concerned with individual statements. There were no relevant guidelines from the institutional review boards of the authors’ institutions regarding the use of publicly available social media data. In the literature, no ethical approval has been taken for using social media data to perform different assessments.

### Methodology

We used social intelligence analysis (SIA) to identify the themes associated with the expression of loneliness. SIA is a broad theme that incorporates multiple social media sources such as Facebook, Reddit, and Quora. SIA is important to gain insights into users’ data and, in our case, understand the dynamics of loneliness. While SIA can be used for a variety of purposes, such as mining content to create stories and finding trends, we used SIA for sentiment analysis of collected data on loneliness. This is a comparative study to assess the themes associated with loneliness across different social media platforms. Therefore, we used Twitter and Reddit to collect data. The following subsections describe in detail the processes of data collection and analysis for Twitter and Reddit data.

### Twitter

**Figure 1:**
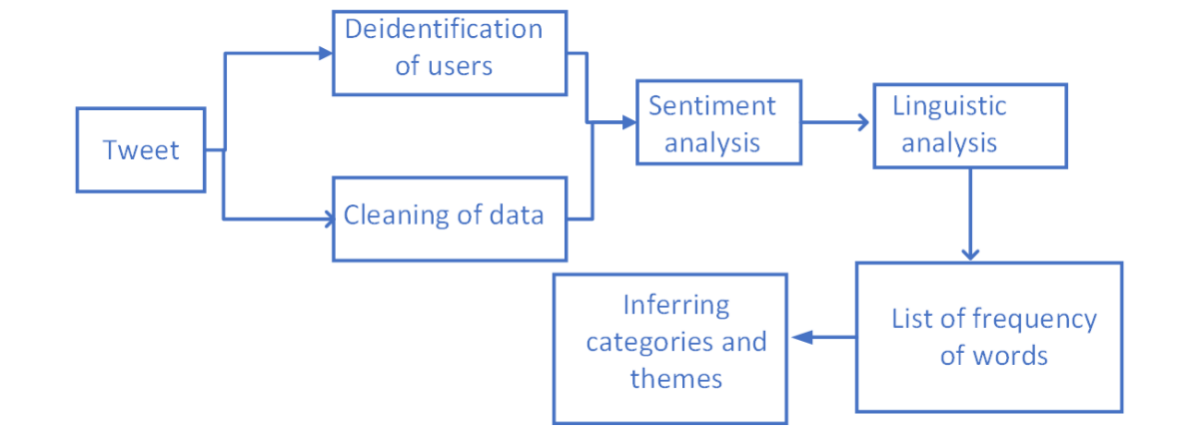
Pipeline for processing Twitter data.

We performed an analysis of publicly available data of users posting about loneliness. Figure 1 presents our pipeline of the analysis of data collected from Twitter. Twitter is a social media platform that is used for connectivity and opinion sharing, and allows users to post via short messages consisting of 280 characters. Twitter provides access to user data through its publicly available Twitter API for developers. Relevant tweets about loneliness were gathered and stored in a database. Twitter data analysis involved the following 3 stages: (1) data collection (tweet collection); (2) division of the collected data into negative and other tweets through preliminary analysis (sentiment analysis of tweets); and (3) further analysis of tweets with negative sentiments through manual coding to find relevant themes and categories (manual coding and analysis of tweets).

#### Tweet Collection

Tweets containing the keywords “lonely,” “loneliness,” “alone,” “isolated,” and “isolation” were collected. In theoretical literature, the words “loneliness” and “lonely” are used to describe the feeling under consideration in this study. The authors of a previous report [21] collected Twitter data based on the keywords “lonely” and “alone.” We went further and included the synonyms and related words of loneliness for collecting our Twitter data.

**Figure 2:**
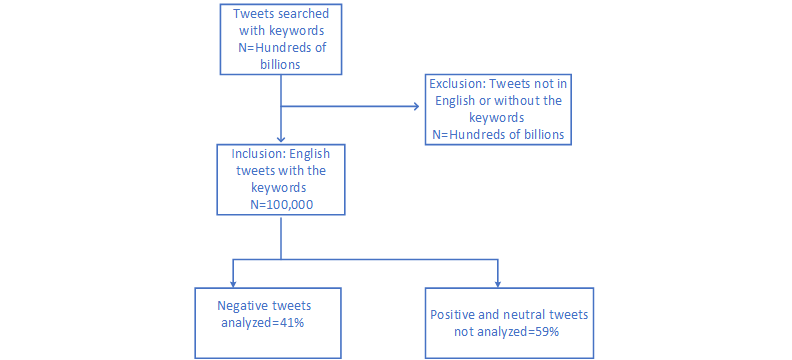
STROBE (Strengthening the Reporting of Observational Studies in Epidemiology) diagram for Twitter data.

We did not want to exhaustively search for data from a specific country because we wanted the collected data to be a proof of concept. We focused exhaustively on cities or countries and collected more data. The majority of tweets were from the United States (38%) and India (24%). The rest were from various countries across the globe. Tweets were extracted from these 2 countries to make a subdataset. This was meant to reflect the majority composition of the dataset. The subdataset contained 100,000 tweets. The collected data were merged based on location, user ID, and tweet ID to identify tweets belonging to the United States and India. We did not have control over the countries from which the data were collected. However, after data collection, we found that most tweets were from the United States and India. From the data collected, we made another dataset that contained 60% data from the United States and 40% from India. No city-wise allocation of data or analysis was carried out. Figure 2 presents the process of collecting data from Twitter and the process of tweet analysis.

#### Sentiment Analysis of Tweets

The next step was to perform a preliminary analysis of the collected tweets through sentiment analysis. This step is required because loneliness is a negative feeling. To determine how it is expressed in its meaning for mental health analysis, we filtered out tweets where the expression of loneliness was negative. If we decided to report all tweets that contained feelings of loneliness, we would not have required a further step. In our case, the problem becomes determining the association of themes (which may represent loneliness) with the keywords depicting loneliness. For instance, we had to assess the relationship of “hurt,” “sick,” “tired,” “sleep,” etc with the expression of loneliness. This task is usually carried out through the association of lexicon categories with tweets including the word “lonely” or “alone.” The collected tweets also contained metaphorical use of lonely or loneliness, which does not pertain to our use of loneliness. Such mentions of loneliness are represented by positive and neutral sentiment tweets. The definition of loneliness in this paper connotates a negative feeling. While loneliness can also be a positive or neutral feeling for some people or some situations, when it comes to its association with mental health issues, the negative consequences of loneliness must be considered.

Therefore, to perform the task of separating tweets with negative connotations for loneliness, we used sentiment analysis. Sentiment analysis was carried out after cleaning the data, such as removing redundant characters, numbers, special characters, user profile IDs, and information like “retweets.” For finding tweets with negative sentiment, we used VADER based on Python’s NLTK. VADER is suited for microblog content, such as that of Twitter. VADER combines the lexicon, that is, dictionary-based analysis, and rule-based approach to characterize the sentiment. VADER uses gold-standard quality like Linguistic Inquiry and Word Count (LIWC) [22], which has been validated by humans. It distinguishes itself from other efficient tools, such as LIWC, in that it is more sensitive to sentiment expression in social media contexts. VADER also provides valence of the sentiment on a range from 1 to 9. Owing to the sentiment score, we can know through VADER the extent to which the sentiment is negative or positive.

This valence is based on generalizable rules that represent grammatical and syntactical conventions that humans use in contexts meant for emphasizing sentiment intensity. For our purpose, another important feature of VADER is the inclusion of sentiment-bearing lexical nonverbal items, such as emoticons, and verbal items, such as slang, acronyms, and initialisms, which are prevalent in social media contexts. The combination of valence polarity through both the lexicon and rule-based approaches is valuable for fine-grained sentiment analysis. The shortcomings of the lexicon-based approach include coverage, general sentiment intensity, and acquiring a new set of human lexical features.

#### Manual Coding and Analysis of Tweets

We stored tweets with negative sentiments separately to carry out further analysis. We assessed the themes and categories that most prominently featured in the tweets with negative sentiment. This we performed through manual coding and analysis. We composed a list where the number of occurrences of each word was presented. Before creating the list, we removed stop words and carried out lemmatization to find out the root words. Lemmatization reduces the number of words, and hence, the list was compact.

Manual analysis of the list of occurrences helped in devising larger socioeconomic or emotional-personal categories. The lists were thoroughly searched to identify meaningful words. There were words used for grammatical construction and others not mentioned significantly. This step was subjective and depended on the number of occurrences in the list. The words to include as topics under different categories were guided by the literature [21,23]. The creation of categories and the assignment of topics were subjective. This part of the classification can be considered qualitative as it was guided by the authors’ judgments and opinions based on the literature.

### Reddit

The Reddit assessment involved only 2 steps: data collection and manual coding. The intermediary step was not required because the Reddit data were forum-based, with a focus on coping with the negative effects of loneliness.

#### Reddit Data Collection

**Figure 3:**
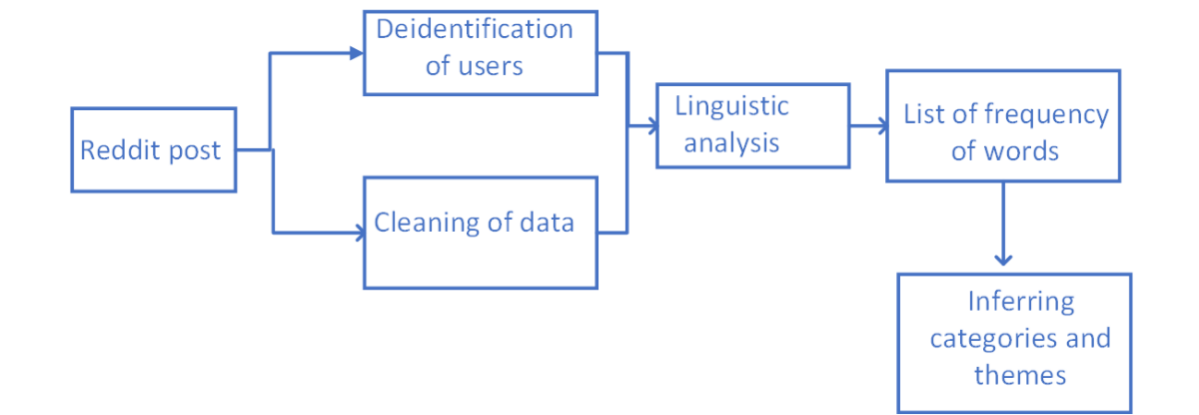
Pipeline for processing Reddit data.

The Reddit data collection methodology was relatively straightforward. Reddit is a forum-based social media platform where people post about a topic on a subforum dedicated to the topic. These subforums are called subreddits. The Reddit API provides access to individual subreddits to download posts along with comments. The API provides access to download the top posts on a topic determined by the number of up-votes and other parameters of engagement. Figure 3 presents our pipeline for processing posts from Reddit. The difference between Figure 1 and Figure 3 is that there is no additional step of sentiment analysis for Reddit posts. After glossing over the subreddit “loneliness,” we found that the posts were about the emotional expression of loneliness and did not involve metaphorical or off-topic use of loneliness. The nature of Reddit and subreddits designed for each topic encourages serious engagement on the topics. Because of this fact, sentiment analysis of Reddit data was not deemed important, and posts were analyzed through the frequency of the occurrence of words to identify the themes and topics most highly associated with loneliness.

Reddit posts from the “loneliness” subreddit were collected through the Reddit API. The “loneliness” subreddit had 13,000 members, and people could post and comment in the subforum. Reddit has its own algorithm for providing a score (ie, higher visibility for posts), which also considers inputs from other users in the form of up-voting. We collected the top 2000 Reddit posts from the “loneliness” subforum with all comments. The comments varied for each post in terms of both number and size. It is worth noting that some of the comments were of the same or even larger size than the original post. Thus, comments can be considered to include data on loneliness. The total number of individual texts analyzed was more than 2000 (posts multiplied by the average number of comments). Some posts did not have any comments, and the maximum number of comments for a post in the data we collected was 55. The average number of comments was 4.51, while the total number of comments was 8570. Combining the comments with the posts provided more than 10,000 unique texts or personal expressions on loneliness from Reddit.

#### Reddit Data Manual Coding and Analysis

We analyzed both the posts and comments to identify the frequency of the occurrence of words to locate the associations of topics and themes with loneliness. The Reddit posts and comments were analyzed without performing any sentiment analysis.

The Reddit posts and comments were collected in a database, and stop words were removed. We performed lemmatization to find out the root words. Then, a list of occurrences of each word was created and analyzed similarly to the method presented in the subsection Manual Coding and Analysis of Tweets.

### Reason for Using Manual Coding and Analysis

As the aim of this study was to identify differences between expressions of loneliness on Twitter and Reddit, assessment of important topics was an objective way to achieve this. A qualitative comparison of different tweets and different Reddit posts could be performed, but this would be biased and limited. When the comparison involves thousands of tweets and Reddit posts, it might not be possible to achieve a meaningful subjective comparison. Breaking down the tweets and Reddit posts into topics and categories allows for objective assessments of similarities and differences.

This method of searching for relevant categories of sociopolitical and personal-emotional content and topics was used because it has more flexibility. Usually, the n-gram method indicates the association of words that co-occur, which cannot be perceived to have happened by chance. However, this method does not indicate the occurrence of individual topics and words, and thus, the impact of a topic will not be known. The use of a keyword-based method (ie, using the collected data for some keywords and then reporting their occurrences) is a deductive approach, and it can help in searching the data for predefined keywords. In this study, we used more of an inductive approach, where we let all the important themes and topics in the data emerge. This allowed us to perform a more thorough analysis of data rather than being limited by keywords to look for in the data. This method to analyze the data is a quantitative approach. We did not qualitatively analyze the tweets and Reddit posts (ie, we did not provide our observations on what is contained in them). We reported the number of occurrences of meaningful words and then divided them into relevant categories. This quantitative stating of sentiment analysis and the words contained in negative sentiment tweets under categories was achieved by reading the literature and following the literature.

## Results

### Comparison of Reddit and Twitter Data

The number of tweets and Reddit posts for analysis was not determined by a specific rule. In the literature, different numbers of tweets and Reddit posts have been used for analysis. For example, Zubaiga [24] carried out an analysis of collected tweets to show the diversity of subjects and dataset sizes. The size of the datasets ranged from over a hundred thousand to more than 10 million. The aim of the study was to reflect in indicative terms the difference in the use of social media according to the expression of loneliness, and therefore, the numbers identified were considered reasonable.

Tables 1 and 2 provide basic details of Twitter and Reddit data, respectively. As discussed earlier, the dataset for Twitter analysis was obtained by combining tweets from the United States and India. The proportions of tweets with negative sentiment for the dataset of each country and for the combined dataset are presented in Table 1. Tweets with negative sentiment were then further analyzed to identify the frequency of the occurrence of topics. For Reddit data, the breakup of the data into words provided a total of more than 25,000 words. For the sake of the meaningful mention of topics and brevity, we only included topics that were mentioned at least 50 times, resulting in 411 words for analysis. Among these 411 words, there were many words related to language construction, such as propositions, and sentence structure. Words that were meaningful in terms of emotions or other expressive qualities were finally included in the analysis.

Table 3 presents the topics highly associated with negative mentions of loneliness in the Twitter dataset. Tweets with negative sentiment were first tokenized and stemmed to obtain a concise list of words and topics associated with loneliness. The list was then analyzed, and meaningful words representing topics of interest, such as emotional, social, and health identifiers, were found. Words like “oh,” “yeah,” and “ur” were ignored when composing the list. For the overall dataset, intimate relationships and interpersonal relationships showed the highest associations with loneliness (Table 3). “Death” as a topic representing matters of health occurred the most in mentions related to loneliness, along with mentions related to COVID. In terms of socioeconomic factors and political expressions, a wide range of topics were identified.

Table 4 provides a list of themes mentioned with loneliness in the loneliness subreddit. The numbers given represent the total number of occurrences of the word or topic in the Reddit posts. The focus was mostly on relations and emotional expression. As longer posts are allowed, people have more space to express their feelings and open up. Social media platforms provide spaces to own one’s vulnerabilities without facing the backlash that can come in the form of social ostracization. In Table 4, the topics and their relevant thematic categories provide deeper insights into the personal-emotional associations of loneliness.

**Table 1:** Analysis of Twitter data.

Country	Sentiment analysis of tweets (negative sentiment proportion)
India	33.8%
United States	48.3%

**Table 2:** Analysis of Reddit data.

Variable	Value, n
Total words	35,057
Words occurring more than 100 times	611
Words occurring more than 1000 times	78

**Table 3:** Topics highly associated with mentions of loneliness in Twitter data.

Thematic area and topic		Mentions, n
**Intimate relationships**		
	Family	2550
	Children	1539
	Cheat	64
	Woman	3534
	Relationship	1213
**Interpersonal relationships**		
	Want	10,452
	Need	9854
	Feel	5173
**Health**		
	COVID	5715
	Die	9652
	Life	3729
**Socioeconomic factors**		
	Injustice	1538
	Money	1344
	Poor	3391
	Culture	4452
	Colorism (black/white)	2276
**Emotional expression/insecurities**		
	Sad	2343
	F*ck	5621
	Hate	3542
**Political**		
	War	1328
	Protect	1198
	Visa	2731
	Modi	1576
	Citizen	543
	Custody	3742
	Protest	4068
**Insomnia**		
	Night	7154
	Day	4315
	Sleep	1564
	Awake	543

**Table 4:** List of themes associated with the expression of loneliness in Reddit data.

Thematic area and topic		Value, n
**Intimate relationships**		
	Love	563
	Women	196
	Relationship	233
	Family	238
	Single	111
	Friends	1015
**Social relations**		
	Friends	172
	Girl	110
	She	587
	Her	467
	People	146
	Online	106
	Meet	237
	Person	428
**Interpersonal relations**		
	Me	2648
	He	101
	Yours	1596
	Us	196
	Everyone	245
	People	1775
	Others	194
**Emotional expression**		
	Thought	197
	Hurt	110
	Trying	284
	Pain	114
	Experience	105
	Remember	101
	Understand	268
	Feeling	435
	Want	941
	Need	539
	Feel	1904
	Wish	229
	Care	262
**Self-focused**		
	I	13,604
	Mental	119
	My	3989
	You	5424
**Work-related**		
	Work	331
	Job	117
	Tried	190
	Time	105
	School	175
**About time**		
	Life	1608
	Year	234
	Live	269
	Old	145

### Similarities and Differences in Reddit and Twitter Data

There are similarities in the categories and themes that can be found in conjunction with mentions of loneliness, but differences in the use of both social media platforms can be crucial in devising strategies to use social media data to understand loneliness. Reddit data are more focused on themes around personal-emotional categories and have a larger number of occurrences of topics around these categories. For Twitter, a range of categories were identified, and personal-emotional categories were the dominant ones, but other topics also occurred. Moreover, the associations of themes and topics with expressions of loneliness were in line with broader psychological linguistic features focused on the self-expression of mental health issues. The difference again was in the range. Twitter can have a wider range of topics that are associated with expressions of loneliness, while Reddit is more focused on psychological personal-emotional categories. These findings will help in providing a guideline for further research into deploying social media data to understand loneliness.

The differences that can be observed in Tables 3 and 4 involve diversity and extensiveness. In Twitter data, the range or diversity of topics and themes can be seen. Because of the limited-character expression on Twitter, people can express their thoughts or opinions, and from these, we can find the association with loneliness. There can be a range of such themes involving direct mentions of loneliness with mentions of a topic in a negative way. On the other hand, Reddit data indicate the extensiveness or depth of a theme or group of topics associated with loneliness.

Some categories, such as “political” and “insomnia,” are missing from Reddit data. The reason for this might be a conjuncture, as direct evidence for the lack of data on Reddit needs to be obtained after following a proper study protocol. However, it can be assumed that because Reddit is more personal (ie, anonymous with the culture being encouraged to reveal personal problems), the general associations of loneliness, such as political and extensive social themes, are not present. For “insomnia,” the easy nature of Twitter (ie, the requirement of short posts) might be seen as a better alternative to discuss sleep problems later at night. Again, these can be conjectural inferences, but the wider social and cultural context can inform the interpretation of these results.

Another reason for Twitter data containing socioeconomic and political categories, which are lacking in Reddit data, is the geographical composition of the data. However, further investigation is needed to determine the geographical composition of the data. According to these findings, Reddit data could be useful for finding the depth of a theme associated with loneliness (ie, what subthemes or topics under a broader category are related to loneliness). Twitter, on the other hand, showed the range and diversity of themes and categories associated with loneliness. This is important to investigate the possible causes of loneliness. The fact that Twitter provides a range of topics and themes and Reddit shows the depth of a theme can be used in a complimentary way.

## Discussion

### Principal Findings

The study performed a comparative analysis of 100,000 tweets and around 10,000 Reddit posts to understand the themes associated with the expression of loneliness according to socioeconomic and personal-emotional topics. The number of Reddit posts was kept lower than the number of tweets because Reddit posts are lengthier. These 2 social media platforms are important because of their unique nature. Twitter allows short texts, while Reddit allows a freehand composition of one’s opinion and analysis. Analysis of the tweets and Reddit posts provided attributes to understand the associations of socioeconomic and personal-emotional factors with loneliness. These attributes included emotion, sentiment, emojis, and topics. The analysis demonstrated that such attributes could help gather evidence and analyze interactions on the topic of loneliness and other such related topics. The first attribute was emotion, which can serve as a guide in understanding people’s reactions. The second most common attribute was relationships. Other thematic areas, such as health, work, self-focused topics, and insomnia-related topics, indicated the intimate nature of loneliness. The major finding of this study was that both social media platforms differed in the range of expressions of themes in association with loneliness.

There were similarities in the themes associated with loneliness on social media, but differences in platform usage are crucial for strategies to understand loneliness. Reddit was heavily focused on personal-emotional themes, with a higher frequency of these topics. Twitter, while also emphasizing personal-emotional themes, included a broader range of categories. The thematic associations with loneliness aligned with broader psychological linguistic features centered on mental health self-expression. The key difference was range. Twitter had a wider variety of associated topics, whereas Reddit concentrated on personal-emotional themes. These findings provide guidelines for future research using social media data to understand loneliness.

The methodology developed in this paper showed the association of loneliness with language, which was associated with mental health issues such as anger and depression. The tweets and Reddit posts analyzed prove that psychosocial linguistic features can be found in the self-expression of loneliness, which can identify the dynamics of loneliness [25,26]. Tweets containing keywords associated with loneliness represent a self-focused discourse, which affirms previous literature on loneliness [27,28].

The topics and categories involve linguistic expressions of underlying mental health and related emotions. The findings of this paper regarding the topics and themes associated with loneliness, including mental health, alcoholism, emotional dysregulation, and sociopolitical circumstances, are backed by the literature. The relationships between mental health issues, such as depression and suicidal ideation, and issues related to neurobiology have been discussed by Lam et al [29]. These include conformity with the literature on the association of loneliness with emotional dysregulation and trouble with relationships [30]. The LIWC was used for different thematic concerns for loneliness by previous authors [23]. The study presented in tabular form the related social and emotional supercategories and relevant subcategories of loneliness. Self-referential pronouns were associated with loneliness according to the study. This study also affirms this finding. Our data present such linguistic categories, which have been proven by previous research to be associated with the linguistic nature of loneliness [31].

Finding themes associated with mental health and other issues in public health, such as loneliness, through social media analysis is interesting because such an analysis can provide insights into the first-hand experiences of people. The primary question in this study was “Is there a significant difference in the use of different platforms when it comes to the expression of loneliness?” The answer to this question is not binary. When it comes to larger patterns of the association of topics with loneliness, both platforms can provide similar patterns. However, the difference lies in the range of topics expressed and the depth of a particular topic. This finding is in line with previous findings. A previous study found that Twitter and Reddit can provide different sentiment expressions after a while for opinions regarding vaccination [32].

Which platform is better to use for the analysis of topics associated with loneliness and other related public health issues? From the data analysis and results, we found that Twitter can provide access to a large number of tweets through its API, but special steps in data cleaning and the combination of different data frames have to be taken in order to carry out a meaningful analysis. Reddit, on the other hand, provides access to lengthier posts where users are free from the constraints of space, and multiple insights into the nature of loneliness can be gained from a single post. Therefore, Twitter provides access to a range of insights, while Reddit provides access to a depth of insights. We also found that Reddit posts, although lesser than tweets, provided a lot more topics, but the topics were constrained by the variety of themes and belonged to similar thematic areas.

This study has provided the following insights into the nature and dynamics of loneliness through the collection of data from different social media platforms (Twitter and Reddit):

1. Despite the limitations of the dataset, the varying nature of loneliness can be observed on both platforms. It gives investigators the opportunity to approach the data on the expression of loneliness on different social media platforms differently.
2. Reddit allows users to share their opinions without limits, and Reddit is built around communities. These communities allow people having similar experiences to share their stories in detail. Therefore, Reddit is more suitable for educational and other messaging services, while Twitter can provide a range of topics, which can further be investigated in depth.
3. The data returned by Twitter can be very large, and to use the data, special data cleaning tools and other steps, such as sentiment analysis, should be used. Reddit data are from communities, and as such, they can be directly analyzed. There can be posts from bots and other such automatic or malicious agents. We did not remove these in this study. These need to be removed before further analysis in future work.

### Limitations

This study has a number of limitations. First, the data from Twitter and Reddit are self-reported and may not represent the broader population. Only English posts were analyzed, limiting generalizability. Future research should explore various platforms and languages for a more comprehensive understanding of loneliness. Second, the dataset size of both Twitter data and Reddit data was small. We used a limited dataset to determine whether loneliness has different expressions on these platforms. The point was not to be exhaustive but indicative. This limitation can be addressed in a future study by increasing the dataset size to verify or change the findings of this study. Third, the Reddit data were not geo-tagged, and thus, we were not able to determine the countries of the users. However, there are methods to identify the locations of the users of Reddit posts. In the future, these methods can be used to carry out a comparative analysis with geo-located Twitter data. Fourth, there was no control over the data returned by the Twitter API regarding specifying a country for data collection. The data returned was not probabilistically sampled, and thus, there may be bias in terms of both content and the country or region from which the data are collected. Fifth, the sentiment analysis of Twitter data may have issues. The automatic classification of tweets into negative and positive through sentiment analysis may not be fully accurate. While this was the basis of the study to carry out automated analysis, the results of this automated sentiment analysis need to be validated by looking at a certain number of tweets that were identified as negative. Current models do not detect some linguistic expressions such as sarcasm and humor. New models can be developed, which can correctly classify the sentiment of tweets that use sarcasm and humor. With this approach, we will be able to know the confidence of the analysis and quantify errors. This is related to the methodology of the study, which did not analyze tweets with negative or neutral sentiments. There is a possibility that even a positive or neutral mention of loneliness is associated negatively with mental health, but for the sake of a coherent analysis of a large number of tweets, this has been removed. Validation of sentiment analysis is needed to determine how many tweets with positive sentiment actually had negative connotations.

### Conclusion

This study aimed to compare data on loneliness from Twitter and Reddit to identify topics and themes associated with the expression of loneliness. The findings of this study suggest that Twitter provides a wide and diverse range of data related to loneliness, while Reddit shows the depth or extensiveness of a theme associated with loneliness. By analyzing the language used in tweets and Reddit posts, it was possible to identify common themes associated with loneliness, such as social isolation, mental health, and relationship issues. The results of this study provide valuable insights into the ways in which people express and experience loneliness on social media platforms. The ability to identify these themes and topics associated with loneliness could be useful for health professionals and researchers to understand the complex nature of loneliness and its impact on individuals.

## Abbreviations

API: application programming interface
LIWC: Linguistic Inquiry and Word Count
NLTK: natural language toolkit
SIA: social intelligence analysis
VADER: valence aware dictionary for sentiment reasoning

## Data Availability

The datasets generated during or analyzed during this study are available from the corresponding author upon reasonable request.
